# Adenovirus Serotype 3 and 7 Infection with Acute Respiratory Failure in Children in Taiwan, 2010–2011

**DOI:** 10.1371/journal.pone.0053614

**Published:** 2013-01-10

**Authors:** Chen-Yin Lai, Chia-Jie Lee, Chun-Yi Lu, Ping-Ing Lee, Pei-Lan Shao, En-Ting Wu, Ching-Chia Wang, Boon-Fatt Tan, Hsin-Yu Chang, Shao-Hsuan Hsia, Jainn-Jim Lin, Luan-Yin Chang, Yhu-Chering Huang, Li-Min Huang, on behalf of the Taiwan Pediatric Infectious Disease Alliance

**Affiliations:** 1 Department of Pediatrics, National Taiwan University Hospital, College of Medicine, National Taiwan University, Taipei, Taiwan; 2 Department of Pediatrics, Chang Gung Memorial Hospital, Taoyuan, Taiwan; Centers for Disease Control and Prevention, United States of America

## Abstract

**Objective:**

Increased incidence of adenovirus infection in children was noticed since September 2010 in Taiwan and severe cases requiring intensive care were noted later. We did this study to find the clinical characteristics and risk factors associated with severe adenovirus infection.

**Patients and Methods:**

We collected cases of severe adenovirus infection between November 2010 and June 2011 to analyze their clinical characteristics in two medical centers in northern Taiwan. Severe adenovirus infection was defined as laboratory-confirmed adenovirus cases with required intensive care. Hexon gene sequencing was performed for molecular genotyping.

**Results:**

45 patients were included, 22 cases (49%) were infected with serotype 7, 19 (42%) with serotype 3, and 4 with serotype 2. The median age (range) was 2.75 years (0.08–15.43 years); 87% were below 5 years. Male to female ratio was 1.65 (28 to 17). Of these patients, 56% had underlying neurological diseases, 50% experienced fever higher than 40°C and 69% suffered fever longer than one week. The clinical diagnosis included pneumonia in 40 (89%) patients, bronchopneumonia in 5 (11%), and encephalitis in 7 (16%). At least 22 patients had pleural effusion. They had complications of respiratory failure (53%), acute respiratory distress syndrome (24%), hypotension (40%), and 6 (13%) patients needed extracorporeal membranous oxygenation. Ten (22%) patients died, all with underlying major systemic diseases and 7 (70%) infected with serotype 7.

**Conclusions:**

Adenovirus serotype 7 and 3 can cause severe disease–even death–in children, especially those with underlying neurological diseases. Patients infected with adenovirus serotype 7 tended to have a higher case-fatality rate.

## Introduction

Adenovirus infection is common in early childhood [1], [2]. The median incubation period is 5.6 days (95% confidence interval 4.8–6.3) [3]. Specific serotypes are often associated with particular clinical syndromes. Adenovirus type 2, 3 and 7 had been associated with severe pneumonia and/or disseminated infection [4]-[6]. Hospital, institutional or community outbreaks were also reported [6]-[8].

An increased number of adenovirus isolated from laboratories of two referral medical centers in northern Taiwan was noticed since September 2010. Then, the rising number of children admitted to the intensive care unit (ICU) for adenovirus infection in these two medical centers was noticed since November 2010. The number did not decrease until July 2011. The nationwide surveillance system of Taiwan also detected a similar trend [9]. In this nationwide surveillance system, an average of 276 respiratory tract specimens from influenza-like illness outpatients, mainly (97%) <18 years of age, were collected each week from 2008 to 2011 and the baseline adenovirus-positive rate in 2008–2010 was 5.75% [9]. The epidemic started in week 11 (March 14) and ended in week 41 (October 16) of 2011 [9]. Mean adenovirus-positive rate during the epidemic was 25.9%, with a peak of 37.3% during week 21 of 2011 [9]. For better understanding of this outbreak, we thus investigated the cases of severe adenovirus infection in these two medical centers between November 2010 and June 2011.

## Patients and Methods

### Study Design and Population

We conducted a study of severe adenovirus infection in children in National Taiwan University Hospital (NTUH) and Chang Gung Memorial Hospital, two medical centers located in northern Taiwan. From November 2010 to June 2011, children with severe adenovirus infections were included in this study. Severe adenovirus infection was defined as children with laboratory-confirmed adenovirus infection by viral culture or polymerase chain reaction (PCR) plus the requirement of intensive care. Viral culture was performed routinely in most children hospitalized for suspicious viral infections in these two medical centers. Real time PCR was performed optionally according to the primary care physician’s clinical suspicion. Hospital-acquired adenovirus infection that was defined as the onset of adenovirus infections occurred 6 days after hospitalization according to the median duration (about 6 days) of incubation period [3].

### Ethics Statement

This study was conducted in Taiwan only, not outside of this country of residence, and the Institutional Review Board approval was obtained form Chang Gung Memorial Hospital (No. 100-2518B). This was a retrospective study without intervention or obtaining clinical specimens and all the data were analyzed anonymously, so informed consent was waived. The waiving of informed consent was also approved by the Institutional Review Board of Chang Gung Memorial Hospital. Every patient had written informed consent for that their data could be used for research under the regulation of the Taiwan law although we did not obtain informed consent for this specific study.

### Clinical Data Collection

Patients’ demographic data, clinical manifestations, intensive care requirement, laboratory results, images, diagnosis, complications and sequelae were collected and analyzed. Respiratory failure was defined as the need of using positive pressure ventilation, which included both invasive modes (e.g., pressure-control ventilation, high frequency oscillatory ventilation) and noninvasive modes (e.g., bilevel positive airway pressure, or continuous positive airway pressure). Acute respiratory distress syndrome (ARDS) was diagnosed if the presentations fulfilled four criteria: 1. acute onset, 2. PaO2/FiO2 less than 200 mmHg, 3. bilateral infiltrates seen on chest radiographs and 4. pulmonary wedge pressure less than 18 mmHg or no clinical evidence of left atrial hypertension, which were defined by American-European Consensus Conference Criteria for Acute Respiratory Distress Syndrome [10]. Diagnosis of disseminated intravascular coagulation (DIC) was based on laboratory data after evaluation with the scoring system proposed by International Society on Thrombosis and Haemostasis [11].

### Molecular Analysis

Hexon gene sequencing was used for serotyping. DNA extraction was performed by using a MagNA Pure LC Total Nucleic Acid Isolation kit (Roche, Germany). For molecular typing, primary PCR primer sets were 5′-TACAACATYGGCTACCAGGG-3′ and 5′-GAGAASGGBGTRCGSAGGTA-3′. Nested PCR was performed with outer PCR products, using primers of 5′-AACTTCCAGCCYATGAG-3′ and 5′-GGRTCCACCTCRAARGTC-3′. The HAdV positive PCR products were purified by gel extraction and were then sequenced using a BigDye Terminator Ready Reaction Cycle Sequencing Kit and an automated sequencer ABI 3730 (Applied Biosystems, Foster City, CA, USA). Obtained sequences were compared with reference sequences of adenoviruses in GenBank using BLAST and the serotypes were determined if the sequences yielded an identity score≧90%.

Because serotype 7 accounted for most cases and deaths in this study, we constructed the phylogenetic tree of adenovirus serotype 7 from 11 cases of severe adenovirus infection and from 3 patients of non-severe adenovirus. The nucleotide sequence homology was inferred from the identity scores obtained using the BLAST program (National Center for Biotechnology Information, Bethesda, MD). A phylogenetic dendrogram was constructed using the neighbor-joining method with the MEGA program 4.0 (Sudnir Kumar, Arizona State University) and the reliability of the tree was estimated with 500 bootstrap pseudo-replicates.

## Results

### Demographic and Clinical Characteristics

**Figure 1:**
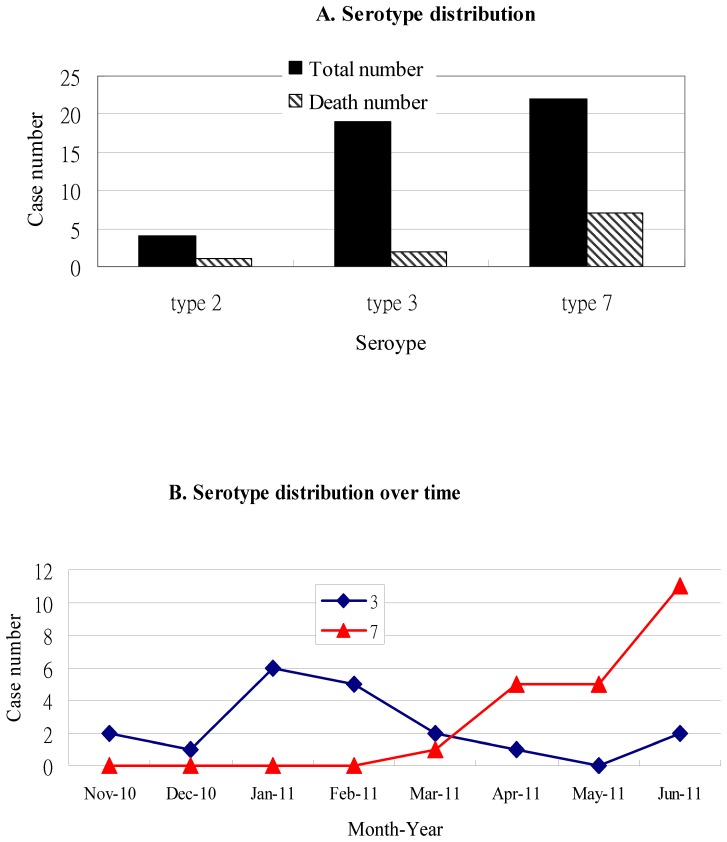
Serotype distribution.

Totally, 45 patients were included. The genotype distribution (Figure 1A) was serotype 7 for 22 cases (49%), serotype 3 for 19 cases (42%) and serotype 2 for 4 cases (9%). This epidemic was initially associated with type 3 and later with type 7 (Figure 1B). Median age (range) was 2.75 years (0.08–15.43 years), and 87% (39/45) of the patients were below 5 years of age. Male to female ratio was 1.65 (28 to 17). More than half of the patients had major underling diseases (Table 1) and both neurological diseases and respiratory diseases played important roles. Of these 45 patients, 19 (42%) were bed-ridden, 21 (47%) had contact and/or cluster history and 11 (24%) had hospital-acquired adenovirus infection.

**Table 1:** Demography and basic characteristics.

	Number (Percentage)
Major underlying diseases	33 (73%)
Neurological disease^1^	25 (56%)
Psychomotor retardation	24 (53%)
Epilepsy	14 (31%)
Respiratory disease^2^	22 (49%)
Cardiovascular disease^3^	3 (7%)
Other major disease^4^	22 (40%)
Bed-ridden	19 (42%)
Contact and/or cluster	21 (47%)
Hospital acquired^6^	11 (24%)

1 In addition to psychomotor retardation and epilepsy, there are also central hypotonia, hypertonia, Miler-Dieker syndrome, metachromic leukodystrophy, schizencephaly, hydrocephalus, brain atrophy and autism.

2 Respiratory diseases included airway anomaly (malacia, stenosis and/or vocal cord palsy, 8 patients), bronchopulmonary dysplasia, asthma, left lung agenesis and emphysema. 5 patients had tracheostomy while 2 used a home ventilator.

3 Most are complex congenital heart disease except one atrial septal defect.

4 Other major systemic diseases included very premature infant (gestational age <32 weeks), chromosome anomaly, acute myeloid leukemia, Pompe disease… etc.

All patients were febrile. The median (range) peak temperature was 39.9°C (38.0–42.0). 95% of patients experienced fever higher than 39°C and 50% experienced fever higher than 40°C. After 10 fatal cases were excluded, the median (range) duration of fever was 10 days (2–30), 69% (24/35) had fever for equal to or over 7 days and 29% (10/35) had fever for equal to or over 14 days. In addition to fever, every patient presented with dyspnea and productive cough. Non-exudative pharyngotonsillitis (85%) and coryza (68%) were also common. 21 patients (47%) had gastrointestinal symptoms including vomiting, diarrhea or abdominal pain. Seizure attack was noticed in 10 patients (22%). Conjunctivitis (7%) and skin rash (9%) were less common.

**Figure 2:**
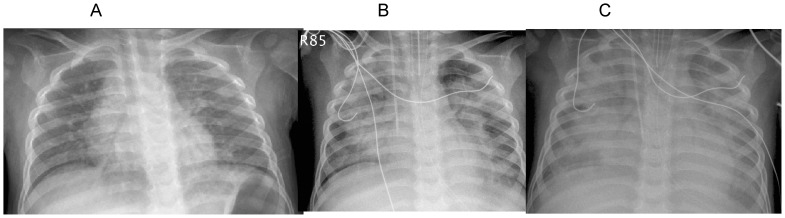
Serial chest radiographs of a 2 year-old boy: 2A shows bilaterally increased infiltration on Day 1 after onset, 2B reveals bilateral patch consolidation on Day 5, and 2C almost complete whiteout of bilateral lungs.

The first chest X-ray taken in most patients revealed increased lung infiltration and/or consolidation bilaterally (Figure 2A). With disease progression, consolidation was more often observed and more extensive (Figure 2B). Bilateral whiteout was seen in 8 (15%) patients (Figure 2C). No cavitating or necrotizing lesion was noted.

### Clinical Diagnosis and ICU Care Requirement

The major diagnosis was pneumonia in 40 (89%) patients and bronchopneumonia in 5 (11%) patients. Encephalitis was diagnosed in 7 (16%) patients by pediatric neurologists according to clinical and electroencephalography findings.

The median hospital stay was 16 days and the median ICU stay was 7 days (Table 2). Respiratory failure with the need of mechanical ventilation and hypotension were the most important indications of ICU care. The median duration of mechanical ventilator use was 12 days while the median duration of inotropic agent use was 4 days. Eleven (24%) patients developed ARDS, high frequency oscillatory ventilation (HFOV) was used in 12 (27%) patients and extracorporeal membrane oxygenation (ECMO) was applied in 6 (13%) patients. Definite or possible DIC was diagnosed in 29 patients because both fibrin degradation product (FDP) and D-dimer noticeably elevated in these patients.

**Table 2:** The clinical characteristics of cases with severe adenovirus infection.

Clinical characteristics	Median duration (range) or percentage (positive number/tested number)
Duration of hospitalization, median (range)	16 days (4–132)
Duration of ICU stay, median (range)	7 days (1–81)
Respiratory failure	53% (24/45)
ARDS	24% (11/45)
Median (range) duration of mechanical ventilation	12 days (3–125)
Usage of HFOV	27% (12/45)
Hypotension	40% (18/45)
Inotropics used	33% (15/45)
Duration, median (range)	4 days (1–60)
Usage of ECMO	13% (6/45)
Duration, median (range)	20.5 days (7–66)
Disseminated intravascular coagulation (DIC)	
Definite	24% (7/29)
Possible	76% (22/29)
CVVH	9% (4/45)

Abbreviations: ICU, intensive care unit;, HFOV, high frequency oscillatory ventilation; ARDS, acute respiratory distress syndrome; ECMO (extracorporeal membrane oxygenation); CVVH (continuous venous-venous hemofiltration).

Chest ultrasonography was used to verify the presence of pleural effusion and was done in 26 patients. Pleural effusion was detected in 85% (22/26) of patients. Pleurocentesis was performed in 16 patients. Chest tube was inserted in 4 patients and median chest tube placement time was 8 days (8–23). Two patients underwent video-assisted thoracoscopic surgery (VATS) but no empyema was noted.

### Microbiological Survey

Samples collected between fever onset and 8 days after defervescence were included for analysis. This time interval was chosen because we found that viral load at 8 days after defervescence was still high in most patients. If PCR was taken as standard, adenovirus was more easily isolated from throat swabs (91%), sputum (88%) and rectal swabs (86%) but with relative difficultly from pleural effusion. The positive isolation rate of pleural effusion was only 39%, although the geometric mean (range) of viral load of pleural effusion was 1.5×10^9^ copies/ml (1.7×10^5^–2.8×10^10^). Lumbar puncture was performed in 9 children if central nervous system involvement was suspected. Cerebrospinal fluid (CSF) culture for both bacteria and viruses was negative in all 9 samples and there was no pleocytosis, elevated protein or decreased glucose in all CSF samples.

Co-infection played a minor role in severe adenovirus infection. There was no definite bacterial co-infection. All patients had negative blood and negative urine culture results for bacteria. Urine pneumococcal antigen test was all negative when it was performed in 29 patients. Possible *Mycoplasma pneumoniae* co-infection was diagnosed in 4 patients according to positive PCR or positive IgM for *Mycoplasma pneumoniae*.

Respiratory virus co-infection was also rare although the study period compassed the respiratory syncytial virus, influenza virus and parainfluenza virus circulating season in Taiwan. Viral antigen tests and PCR were performed frequently. There was only one case with positive influenza A PCR test. One throat swab yielded adenovirus and parainfluenza virus simultaneously. Only one patient had positive sputum respiratory syncytial virus antigen and the other one had positive Chlamydia antigen, respectively. 21 patients (47%) had gastrointestinal symptoms. However, only 2 patients had positive stool norovirus antigen and there was no positive stool rotavirus antigen.

### Clinical Laboratory Parameters

Clinical laboratory data were collected between fever onset and 8 days after defervescence for analysis; Table 3 shows the results. Pleural effusion was grossly bloody in 44% (7/16) of patients and had extremely high lactate dehydrogenase (LDH), elevated protein level but normal glucose (Table 3) and pleocytosis (the median white cell count 1522/µL and the median lymphocyte percentage was 61%). Leukopenia and/or thrombocytopenia were seen in more than 60% of patients. Nevertheless, during serial follow up, both leukocytosis and leukopenia could be detected in the same patient. Left shift (>5% proportion of immature neutrophil cells) [12] was noted in 42% of patients. Leukocytosis with left shift was usually noted in the early course while leucopenia and thrombocytopenia were most often found when disease severity progressed. Like pleural effusion, extremely high serum LDH was noted. Liver enzyme elevation was more prominent for aspartate aminotransferase (AST). Elevated creatinine kinase, hypoalbuminemia and hyponatremia were also characteristic.

**Table 3:** Laboratory data of cases with severe adenovirus infection.

Data	Percentage (positive number/tested number) or Median (range)
Pleural effusion	
Grossly bloody	44% (7/16)
White blood cell count	1522/µl (19–9500)
Lymphocyte	61% (12–100)
Neutrophil	4% (0–84)^1^
Mesothelial cell and histiocyte	28% (0–81)^1^
Glucose	107 mg/dL (31–118)
Total protein	3.5 g/dL (2.4–5.4)
Lactate dehydrogenase	7853 U/L (1845–21530)
Complete blood cell count	
Platelet	
Initial	239 K/µL (34 K–496 K)
Lowest	108.5 K/µL (7–438 K)
<150 K/µL	64% (29/45)
White blood cell	
Initial	8900/µL (2930–33900)
Highest	12000/µL (3440–39690)
Lowest	4400/µL (700–16060)
Leukopenia (<5000/µL)	60% (27/45)
Biochemistry	
Lactate dehydrogenase	2048 U/L
>500 U/L	95% (18/19)
Aspartate aminotransferase	164 U/L (21.0–3520.0)
>55 U/L	79% (34/43)
Alanine aminotransferase	53 U/L
>45 U/L	60% (25/42)
Creatinine kinase	276 U/L (20–61740)
>130 U/L	82% (23/28)
Albumin	2.8 g/dL (1.9–4.6)
<3.5 g/dL	76% (28/37)
Sodium	132 (119–143)
<135 mEq/L	64% (27/44)
Creatinine	0.55 mg/dL (0.22–6.27)
>1 mg/dL	15% (6/39)
C-reactive protein	
Initial	3.4 mg/dL (0.4–37.7)
Highest	7.46 mg/dL (0.6–37.7)

### Outcome

10 (22%) patients died and they all had major underlying diseases. 7 (70%) fatal cases were infected with serotype 7 (Figure 1A). The median duration from disease onset to death was 25 days (range from 4 to 71 days). Most deaths were associated with acute respiratory distress syndrome.

Complication occurred in 24%. Except 1 with pneumothorax and 3 with asthma attacks, 6 patients had nosocomial infection (including ventilator associated pneumonia, infected pressure sore, and central venous catheter associated infection), 4 patients had ECMO-related complications, including thrombus and aneurysm formation, and encephalopathy after prolonged sedation was diagnosed in 2 patients.

If survived, 77% (27/35) had full recovery. 4 patients had neurological sequelae, including epilepsy, muscle weakness and developmental regression and another 4 patients had respiratory sequelae: 2 of them were bronchiolitis obliterans and the other 2 needed home BiPAP use.

### Molecular Analysis

**Figure 3:**
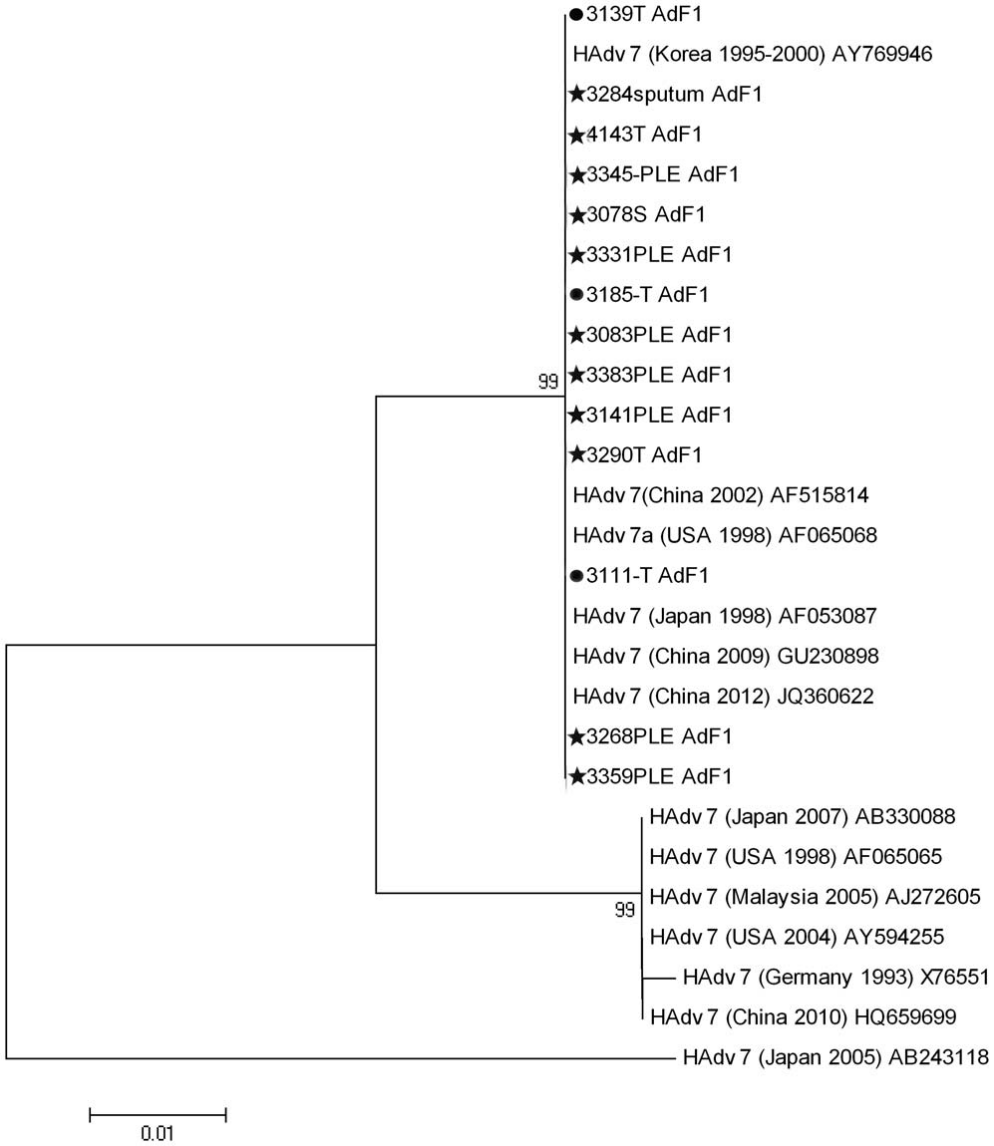
Phylogenetic tree of the hexon sequences for 11 severe adenovirus serotype 7 infection samples (marked with ★), 3 non-severe adenovirus serotype 7 infection samples (marked with •) and reference sequences from other countries in different years. The sites of the studied strains were from T, throat swabs, PLE, pleural effusion, and S, stool. The serotype 7 sequences of this study shared 100% homology with the strains from America, Japan, Korea and China, and one of the reference strains, human adenovirus 7 strain 0901/HZ/ShX/CHN/2009, caused a severe lower respiratory tract disease outbreak in infants in Shaanxi Province, China recently.

We compared the hexon sequences of 11 severe adenovirus serotype 7 infection samples and 6 non-severe adenovirus serotype 7 infection samples. They are all very similar. Only two samples had one and three different nucleotides, respectively. After translated to amino acid, we only observed one sample to have one amino acid change (Histidine to Asparagine). Phylogenetic tree was presented in Figure 3. Our serotype 7 sequences shared 100% homology with the strains from America, Japan, Korea and China, and one of them, human adenovirus 7 strain 0901/HZ/ShX/CHN/2009 (GenBank GU230989), caused a severe lower respiratory tract disease outbreak in infants in Shaanxi Province, China recently [7].

## Discussion

We reported a severe adenovirus infection epidemic in northern Taiwan, which was caused by both serotype 3 and serotype 7. Most of these severe adenovirus cases had pleural effusion, acute respiratory failure, and about half of them had hypotension. These severe cases usually have underlying diseases, especially neurological diseases.

Noticeably elevated LDH in both serum and pleural effusion was characteristic laboratory findings. Elevated liver enzymes and hypoalbuminemia were common, too. The kidney was relatively spared. In Peled, N., et al’s study, high levels of serum LDH and AST were correlated with the severity of adenovirus infection as defined by duration of hospitalization. LDH level correlated statistically with high AST and low oxygen saturation, and thus suggested that the main source of increased LDH might be from the liver and lungs [13]. In addition, the bloody appearance and high LDH in pleural effusion indicated the intensity of lung damage just as elevated liver enzyme and hypoalbuminemia reflected liver injury. The lung and liver insults were key components in pathogenesis of severe adenovirus infection.

Over fifty serotypes of adenoviruses are pathogenic in humans but often cause mild and self-limited disease. Different serotypes may display different tissue tropisms and correlate with clinical manifestations of infection. Some serotypes are reported to cause severe diseases, especially type 3, 4, 7, and 21. Among them, adenovirus serotype 7 was associated with the most severe clinical course and resultant long-term pulmonary sequelae [14]. In Taiwan, several outbreaks of adenovirus were reported and most outbreaks were caused by serotype 3, with severe disease entities and even death [15]-[18]. During November, 1999 to March, 2000, there was an outbreak related to serotype 3 and serotype 7 in Southern Taiwan [19]. This was the only reported cluster of adenovirus serotype 7 before 2011, but no severe cases were mentioned [19]. Between 2004 and 2005, serotype 3 accounted for another outbreak in northern Taiwan [15], [18]. Two medical centers did further serotype identification during this period and found serotype 3 accounted for 74.6% to 87.2% of circulating adenovirus [15], [18]. Additionally, according to the nationwide surveillance data from the Centers for Diseases Control of Taiwan, genotype 3 accounted for 35% to 58% of yearly adenovirus isolates selected for genotyping analysis from 2008 to 2010 [9].

In this study, both serotype 3 and serotype 7 were involved in this outbreak, but fatal cases were more frequently infected with serotype 7 (70%). The mortality rate for serotype 7 and non-serotype 7 were 32% (7 out of 22) and 14% (3 out of 22), respectively. Although the mortality rate caused by serotype 7 was higher than that caused by non-serotype 7, the difference did not reach statistical significance (p = 0.13). The results may be influenced by the small sample size. For this outbreak in Taiwan, Tsung TP et al reported serotype 3 was the predominant strain (74%) and serotype 7 represented for 10% of influenza-like illness outpatients based on the nationwide surveillance system [9]. However, serotype 7 was significantly associated with severe diseases because it accounted for 10%, 12%, and 41% of infections among outpatients, inpatients with nonsevere infection, and inpatients with severe infection, respectively (p<0.01) [9]. Several previous studies also reported that serotype 7 was associated with severe clinical presentation and worse outcome [7], [8], [14]. Therefore, public health authority should warn people if the circulating serotype is serotype 7.

The underlying diseases are very important risk factors of severe adenovirus infection. Adenovirus type 7 was reported to cause outbreaks of infection in institutions where severely disabled children developed life-threatening disease [8]. In our study, most of the severe cases were also disabled children with severe underlying diseases, especially neurological underlying diseases, and all the fatal cases had systemic underlying diseases. Neurologically disabled children may be more prone to develop respiratory failure or multiple organ failure after adenovirus infection. Similar observation was also noted in neurologically disabled children with pandemic H1N1 influenza infection: among the 24 fatal children with high-risk medical conditions, 22 (92%) had neurodevelopmental conditions, e.g. developmental delay or cerebral palsy [20].

In conclusion, severe adenovirus pneumonia was associated with serotype 3 and 7 during the 2010–2011 outbreak in Taiwan and it usually occurred in cases with underlying neurological disability.
